# Multisensory stimuli improve relative localisation judgments compared to unisensory auditory or visual stimuli

**DOI:** 10.1121/1.5042759

**Published:** 2018-06-25

**Authors:** Laura C. A. Freeman, Katherine C. Wood, Jennifer K. Bizley

**Affiliations:** Ear Institute, University College London, 332 Gray's Inn Road, London, WC1X 8EE, United Kingdom laura.freeman89@hotmail.co.uk, katherinecwood@gmail.com, j.bizley@ucl.ac.uk

## Abstract

Observers performed a relative localisation task in which they reported whether the second of two sequentially presented signals occurred to the left or right of the first. Stimuli were detectability-matched auditory, visual, or auditory-visual signals and the goal was to compare changes in performance with eccentricity across modalities. Visual performance was superior to auditory at the midline, but inferior in the periphery, while auditory-visual performance exceeded both at all locations. No such advantage was seen when performance for auditory-only trials was contrasted with trials in which the first stimulus was auditory-visual and the second auditory only.

## 1. Introduction

Both auditory (Mills, 1958; Makous and Middlebrooks, 1990; Charbonneau *et al.*, 2013; Wood and Bizley, 2015; Carlile *et al.*, 2016) and visual localisation acuity declines with eccentricity (Mateeff and Gourevich, 1984; Perrott *et al.*, 1993; Charbonneau *et al.*, 2013). Few studies have attempted to directly compare spatial acuity for auditory and visual stimuli throughout the visual field and focus instead on the spatial capture observed when spatially separated auditory-visual signals are presented (Howard and Templeton, 1966; Bertelson and Radeau, 1981). Two exceptions to this are Perrot *et al.* (1993) and Charbonneau *et al.* (2013). Both determined that both visual and auditory localisation judgments declined as stimuli move from central to peripheral space. However, the studies produced conflicting results, and neither study perceptually matched stimuli across modalities. Perrott *et al.* (1993) did not test bimodal stimuli, but reported equivalent auditory and visual performance, while Charbonneau *et al.* (2013) reported superior visual performance and no advantage for auditory-visual stimuli. However, in their study on every trial an auditory-visual reference was provided and only the target varied in modality complicating comparisons with unisensory performance.

Since both visual contrast (Kanai *et al.*, 2004) and auditory signal-to-noise ratio impact upon localisation accuracy (Wood and Bizley 2015) our goal was to present perceptually matched stimuli so that localisation acuity could be directly compared across modalities. The aims of this study were therefore to determine (i) how relative localisation judgments vary throughout frontal space for *equally detectable* auditory and visual signals and (ii) whether an auditory-visual signal conferred a processing advantage over the most effective unisensory stimulus. Finally, because we observed a clear multisensory benefit, in experiment 2 we tested stimuli in which an auditory-visual reference was followed by an auditory only target. It was hypothesised that when comparing the ability to make auditory and visual relative localisation judgments with perceptually matched stimuli, visual performance would exceed auditory in central locations (i.e., at the fovea). However, visual localisation acuity declines linearly with eccentricity (Michel and Geisler, 2011), whereas the decline in auditory localisation cues is more modest with cues remaining robust across a range of eccentricities (Macpherson and Middlebrooks, 2002; Wood and Bizley, 2015). We therefore predicted that at more peripheral locations auditory relative localisation judgments might be more accurate than visual.

## 2. Methods

### 2.1 Participants

This experiment received ethical approval from the UCL Research Ethics Committee (3865/001). 14 self-reported normal hearing adults with normal or corrected-to-normal vision, between the ages of 18 and 35 participated in experiment 1. Two participants were excluded due to poor performance [average sensitivity score (*d*′) < 0.5]. Nine of the remaining 12 participants participated in experiment 2.

### 2.2 Procedure

Before embarking on the main experiment(s) participants performed two short threshold tests which measured auditory and visual performance at a range of signal levels in the presence of background noise so that detectability could be matched across modalities. The thresholds from these tests (see below for threshold estimation procedures) were then used to set the signal levels for the main experiments. In all experiments participants performed a two-interval forced choice task by comparing sequentially presented reference and target stimuli and determining whether the target originated from the left or right of the preceding reference. The goal of the first experiment was to measure the accuracy with which relative localisation judgments could be made throughout the frontal hemifield when the stimuli were either auditory, visual or auditory-visual. Reference and target pairs were always separated by 15°. In experiment 2 we contrasted auditory performance with trials in which there was an auditory-visual reference stimuli in an otherwise identical procedure.

### 2.3 Testing chamber

**Fig. 1.**
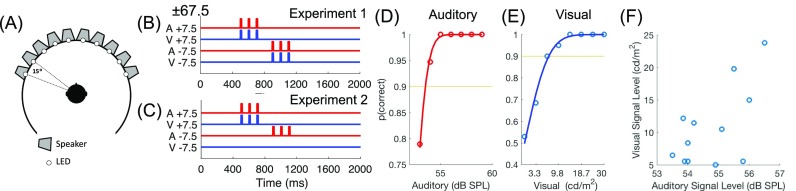
(Color online) (A) Schematic of the testing chamber; (B), (C) schematic of the trial structure for experiment 1 [(D), AV trial] and experiment 2 [(E), AV reference trial] showing for one example trial in which the relative location of the stimulus shifts leftwards from +7.5° to −7.5°. Example threshold functions for auditory (D) and visual (E) detection abilities. (F) Auditory and visual signal levels for all participants.

For testing, participants sat in the middle of an anechoic chamber surrounded by a ring speakers arranged at 15° intervals from −67.5° to +67.5° [Fig. 1(A)]. Stimuli were presented by Canton Plus XS.2 speakers (Computers Unlimited, London) and white light emitting diodes (LEDs), mounted below each speaker, via a MOTU 24 I/O analogue device (MOTU, MA). For auditory stimuli the MOTU output was amplified via 2 Knoll MA1250 amplifiers (Knoll Systems, WA). Both the speakers and LEDs were visible to participants. The participants' heads were kept in a stationary position and supported there by a chin rest. Participants were asked to maintain visual fixation on a fixation cross located on the speaker ring at 0° azimuth. Head and eye position were remotely monitored with an infra-red camera to confirm that subjects did not make deliberate or reflexive orienting movements to the reference stimuli.

### 2.4 Stimuli

All stimuli were generated in matlab and presented using the psychtoolbox extension (Brainard, 1997) at a sampling frequency of 48 kHz. Identical stimuli were used for the threshold test and experiments 1 and 2. In the auditory (A) condition, three pulses of white noise were presented from a reference speaker, followed by three pulses of white noise from a target speaker. In the visual (V) condition, three pulses of light were emitted from a reference LED mounted on a speaker, followed by three pulses of light from a target location. In the auditory-visual (AV) condition in experiment 1 spatially and temporally coincident light and sound pulses were presented [Fig. 1(D)]. In experiment 2, spatially and temporally coincident sound and lights were presented at the reference location, and only the auditory stimulus was presented at the target location [Fig. 1(E)]. Stimulus pulses were identical to those used in Wood and Bizley (2015): 15 ms in duration, cosine ramped with 5 ms duration at the onset and offset of each pulse. Pulses were presented at a rate of 10 Hz with a 185 ms delay between the end of the final pulse at the reference speaker and the first pulse at the target speaker in order to aid perceptual segregation of the reference and the target. The pulses were embedded in a noisy background comprised of independently generated auditory and visual noise from each speaker/LED. The amplitude of the noise was varied independently at each location every 15 ms with amplitude values drawn from a distribution whose mean and variance could be controlled (as in Wood and Bizley, 2015). Values were drawn from a Gaussian distribution with a mean level at each speaker of 49 dB sound pressure level (SPL) and a standard deviation of 1.5 dB SPL giving a mean noise level across all speakers of 63 dB SPL (calibrated using a Bruel and Kjær 3110–003 measuring amplifier placed at the centre of the speaker ring). Visual noise was generated in the same way with an average background level of 0.2 cd/m^2^ and a standard deviation of 0.2 cd/m^2^. Luminance was measured with a Konica-Minolta CS-100 A luminance meter from the centre of the speaker ring. This temporal structure served to promote the perception of multiple spatially separated sources rather than a single diffuse noise source. At the start of each trial the noisy background was ramped on with a linear ramp over 1 s and ramped down over 1 s at the end of the trial. The stimulus pulses, which constituted the reference and target, were presented at an unpredictable interval 50–1000 ms after the noise reached its full level, drawn from a uniform distribution from 50 to 1000 ms in 50 ms steps, pseudorandomised across trials.

### 2.5 Threshold

Participants were oriented to face a speaker at the frontal midline (0° azimuth). The reference stimulus was always presented from this speaker/LED, and the target was presented from a speaker/LED at either −60° or +60°. Auditory and visual stimuli were presented in separate testing blocks. Participants reported the direction in which the stimulus moved using the left and right arrows on a keyboard to indicate −60° and +60°, respectively. Auditory stimuli were presented at ten different SNRs by varying the signal attenuation in 1 dB steps over a 10 dB range from 53 to 63 dB, and visual stimuli were presented at ten SNRs by varying voltage values driving the LEDs from 1.4 to 30 cd/m^2^. Percentage correct scores for left/right judgments were fit using binomial logistic regression [Figs. 1(D) and 1(E)] and the signal value at a threshold of 90% correct was extracted from the fitted function. The aim was to present stimuli at a level that was clearly audible/visible, but difficult enough to be challenging for the subsequent relative localisation task. The threshold therefore served both to match difficulty across participants and sensory modalities. The resulting signal attenuation values for all 12 participants are shown in Fig. 1(F).

### 2.6 Experiments 1 and 2

In experiments 1 and 2 participants were oriented such that they faced a fixation light placed between the front two speakers [such that the speakers closest to the midline were at ±7.5°, Fig. 1(A)]. The signal attenuations were fixed at the levels determined by the threshold test [Fig. 1(F)]. Reference and target sounds were always separated by 15°, with reference and target stimuli being presented throughout the frontal ±67.5°. As in the threshold test, participants made left/right decisions via the arrow keys on a keyboard. Trials were initiated automatically after the previous response was registered and were divided into 5 min testing blocks, between which participants were free to take a break. Experiments 1 and 2 took approximately 40 and 30 min to complete, respectively. In experiment 1 auditory, visual, and spatially and temporally coherent auditory-visual stimuli were presented [Fig. 1(B)], in experiment 2 auditory stimuli and stimuli in which a spatially and temporally coherent auditory-visual reference was presented, followed by an auditory target.

### 2.7 Analysis

Overall performance was assessed using signal detection theory to calculate sensitivity index (*d′*) statistics for participants' ability to discriminate whether a target sound moved left or right, with hits being (arbitrarily) defined as rightwards choices for rightwards moving stimuli, and false alarms (FAs) being defined as right choices for leftwards moving stimuli (Green and Swets, 1966):

d′=Z(Hit)−Z(FA), (1)

where Z(p) is the inverse cumulative distribution function of the Gaussian distribution. Performance was estimated across reference-target pairs of the same locations (so that the change in localisation cues for left moving and right moving trials were equivalent) and considered relative to the mean location of that speaker pair.

Bias was calculated such that negative numbers indicate a bias to rightwards choices (Macmillan and Creelman, 1991),

$$Bias=-\frac{Z(Hit)+Z(FA)}{2}.$$ (2)

Multisensory gain (i.e., the benefit provided by a redundant cross-modal stimulus) was calculated as the improvement in performance in the multisensory condition relative to the best unisensory condition (in experiment 1) or the unisensory auditory stimulus (in experiment 2). Since performance varied with azimuthal position, values were expressed as a % relative to the best unisensory performance for that eccentricity (Charbonneau *et al.*, 2013). Reaction times were extracted relative to the onset of the first stimulus, and compared to predictions of the race model in order to determine whether any reaction time gain was faster than would be anticipated by two independent processes (Miller, 1982; Ulrich *et al.*, 2007). Group level statistical analysis was performed in SPSS (v24, IBM) using repeated measures analysis of variance (ANOVA). Two-way repeated measures ANOVAs were performed to determine the impact of modality and spatial location on sensitivity, bias, and reaction time measures. One-way repeated measures ANOVA was used to determine the impact of eccentricity on multisensory gain or location within a modality.

## 3. Results

Before participating in experiment 1 listeners performed two short threshold tests (see Sec. 2.5). These served to match the detectability of signals across modalities by assessing performance across a range of signal attenuations [Figs. 1(B) and 1(C)]. This step was critical as it allowed us to test each modality at an equivalently difficult level so that we could directly compare localisation ability across auditory and visual signals, it further serves to match difficulty across participants.

### 3.1 Experiment 1

**Fig. 2.**
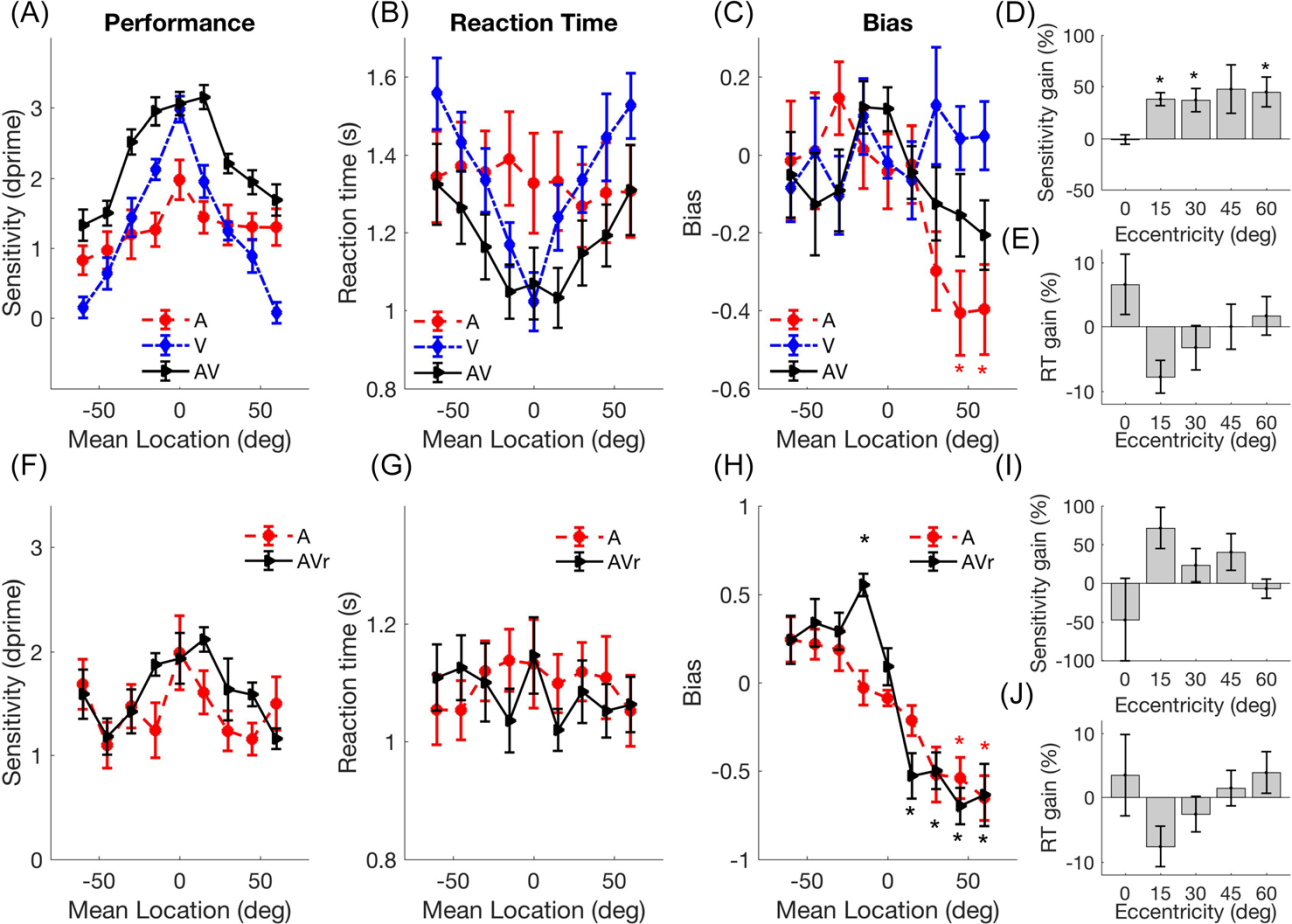
(Color online) Mean (±SEM) (A) *d*′ scores for A, V, and AV trials as a function of the mean reference-target location, (B) reaction times, (C) bias, (D) sensitivity gain (% gain relative to best unisensory performance), (E) reaction time gain (% relative to fastest unisensory) for experiment 1. Asterisks indicate values are significantly non-zero (p < 0.05 corrected for five comparisons). (F)–(J), as (A)–(E), but for experiment 2.

Experiment 1 tested the ability of listeners to perform relative localisation judgments with A, V, or spatially and temporally coincident AV signals, presented at their pre-determined signal attenuations. Performance varied throughout azimuthal space [Fig. 2(A)] with the best performance being obtained for stimuli close to the midline, and performance dropping off at more lateral locations. V performance, although superior to A at the midline, dropped with eccentricity more dramatically such that A performance was superior in the periphery. AV performance exceeded A and V at all locations except for stimuli crossing the midline, where performance was close to ceiling for both V and AV stimuli. Both stimulus modality (F_(2,22)_ = 20.8, p = 0.0006) and location (F_(8,88)_ = 24.9, p = 1.25e–19) influenced *d′*, with a significant modality × location interaction (F_(16,176)_ = 20.8, p = 1.0934e–9). Pairwise *post hoc* comparisons revealed that AV performance was significantly different from both A and V (which were statistically indistinguishable) and that central reference locations were significantly different from peripheral ones (Table 1).

**Table 1.** Post-hoc pairwise comparisons (Bonferoni corrected) for the effect of spatial position in experiment 1. Grey squares indicate significant differences (p < 0.05).

Mean Location	−60	−45	−30	−15	0	15	30	45	60
Experiment 1	−30, −15, 0, 15, 30	−30, −15, 0, 15	−60, −45	−60, −45, 30, 45, 60	−60, −45, 30, 45, 60	−60, −45, 15, 60	−60, −15, 0, 15, 60	0	−15, 0, 15, 30, 45

Multisensory gain was calculated by comparing *d′* values obtained in the AV condition with those in the best unisensory condition, with data folded across space to determine how eccentricity impacted multisensory gain [Fig. 2(D)]. T-tests (Bonferoni corrected for five locations) indicated that multisensory gains were non-zero at 15°, 30°, and 60° (p < 0.01) and gain did not vary significantly with eccentricity (effect of eccentricity on multisensory gain: F_(4,44)_ = 1.82, p = 0.142).

Reaction time measures [Fig. 2(B)] for relative localisation judgments with A and V stimuli showed distinct patterns: V reaction times rose monotonically with increasing eccentricity (one way ANOVA of location on V reaction times F_(8,88)_ = 16.1, p < 0.001), while A reaction times were consistent across space (F_(8,88)_ = 0.85, p = 0.57). AV reaction times showed an intermediary pattern of variability increasing more gradually with eccentricity (AV: F_(8,88)_ = 6.94, p < 0.001) and, with the exception of the central location, always being faster than either modality alone. A two-way ANOVA investigating the influence of position and modality on reaction time revealed effects of both location (F_(8,88)_ = 10.34, p = 4.3405e–10) and modality (F_(2,22)_ = 4.46, p = 0.024) with a significant modality × location interaction (F_(16,176_) = 5.73, p = 6.7686e–10). *Post hoc* analysis revealed that AV reaction times were significantly faster than both auditory and visual reaction times. While AV reaction times were significantly faster than either modality alone, they did not violate the race-model (Miller, 1982; Ulrich *et al.*, 2007) (p > 0.05 at all locations). Moreover, when reaction times were expressed as multisensory gain [Figs. 2(D) and 2(E)], no location had a significantly non-zero gain (t-test against zero, Bonferoni corrected p < 0.01).

Bias measures were calculated for performance in each modality [Fig. 2(C)]. For both V and AV trials bias was constant across space [one way repeated measures ANOVA, AV: F_(8,88)_ = 1.27, p = 0.270 V: F_(8,88)_ = 0.64, p = 0.742] whereas for A bias was influenced by spatial position (F_(8,88)_ = 2.92, p = 0.006), with bias values indicating that participants were more likely to report outward moving stimuli at peripheral locations. Consistent with this, a two-way repeated measures ANOVA directly comparing these values revealed no effect of either modality (F_(2,22)_ = 2.76, p = 0.085) or spatial position (F_(8,88)_ = 1.279, p = 0.269), but a significant modality × position interaction [F_(16,176)_ = 2.23, p = 0.006; Fig. 2(C)]. In summary, AV stimuli conveyed an advantage in both performance and reaction time compared with the best unisensory stimulus, throughout frontal space.

### 3.2 Experiment 2

Experiment 2 aimed to determine whether the improvement in relative localisation ability for a AV stimuli could be observed by presenting an AV reference stimulus and an auditory-only target. Nine of the 12 participants from experiment 1 performed experiment 2, which included trials which were A-only for both reference and target, and AV reference A-target trials. An AV reference provided no advantage over an A reference when the target was A alone [Fig. 1(E)]: Performance varied weakly with reference location (F_(8,64)_ = 2.391, p = 0.025, *post hoc* pairwise comparisons all p > 0.05), but not modality (F_(1,8)_ = 2.56, p = 0.148), nor was there a significant modality × location interaction [F_(8,64)_ = 1.788, p = 0.096; Fig. 2(F)]. Reaction times were also uninfluenced by reference modality [spatial position: F_(8,64)_ = 1.06, p = 0.5; modality: F_(1,8)_ = 1.179, p = 0.309; Fig. 2(G)]. Consistent with an AV reference offering no perceptual advantage, measures of multisensory gain were not significantly different from zero [t-test, all p > 0.05, corrected for five comparisons; Figs. 2(I), 2(J)]. Finally both A and AV reference conditions showed very similar patterns of bias, with listeners tending to show preference to respond away from the midline [spatial position: F_(8,64)_ = 16.46, p = 0.000; modality: F_(1,8)_ = 1.179, p = 0.309; modality × position interaction: F_(8,64)_ = 3.43, p = 0.002; Fig. 2(H)]. Thus the multisensory enhancement seen in experiment 1 required that both stimulus intervals contained a multisensory stimulus.

## 4. Discussion

In these experiments we tested the accuracy with which observers could discriminate 15° shifts in location between sequentially presented reference and target stimuli. Difficulty matched auditory and visual stimuli were used so that performance could be directly compared across modalities. Visual acuity was highest for central locations and fell off sharply at more peripheral locations. Auditory acuity was highest at the midline, and also declined at more peripheral locations. However, the change in auditory relative localisation ability with eccentricity was much smaller in magnitude (Δ*d′* of 1.2 for A, compared to Δ*d′ = *2.9 for V) than for visual ability. Performance for auditory-visual stimuli also varied throughout space and, except at the midline where performance matched V (and performance was at or close to ceiling), was better than either A or V at all locations. AV stimuli were processed faster than A or V alone. Consistent with previous studies (Charbonneau *et al.*, 2013), V reaction times increased with eccentricity and AV reaction times mirrored these, whereas processing time was not contingent on eccentricity for A-only stimuli.

Our signal detection analysis demonstrated that while auditory acuity was higher than visual acuity in the periphery, participants were significantly biased towards reporting movements away from the midline for auditory, but not auditory-visual or visual judgments and that this tendency was particularly marked for stimuli on the right side of space. The eccentricity of both auditory (Mateeff and Hohnsbein, 1988; Ihlefeld and Shinn-Cunningham, 2011) and visual (Mateeff and Gourevich, 1983; Fortenbaugh and Robertson, 2011) signals tends to be underestimated at more peripheral locations; this potentially offers an explanation for why outward judgments were favoured, but suggests either this effect is more marked for sound localisation, or this factor does not underlie the pattern of auditory bias observed.

These results emphasise that the advantage conferred by visual stimuli exists only in central regions closest to the fovea; at more lateral locations auditory stimuli are more accurately localised. However, integrating stimuli offers an advantage throughout space. These findings mirror those of Perrott *et al.* (1993); although they demonstrated no statistical difference between auditory and visual stimuli, the group data for their four observers suggest that visual acuity exceeded that of auditory acuity at 0° (minimum visual angle, MVA = 0.5°, minimum auditory angle, MAA = 1°), was equivalent (roughly 2°) at 20°, and auditory acuity exceeded visual acuity at more lateral locations (for example, at 80° reference MAA = 4°, MVA = 7°). Charbonneau *et al.* (2013) performed a similar experiment to the present study, except that they only varied the modality of the target stimulus: a spatially congruent AV reference was presented on every trial. They reported that AV performance matched that of V, and exceeded A, at all locations. The difference in the results presented here and those in Charbonneau *et al.* (2013) is likely explained by our presenting matched-detectability stimuli across modalities which provided the opportunity to make direct comparisons in spatial acuity. Our data are consistent with previous reports that saccades made to AV targets are both faster and more accurate than to either modality alone—with saccades to unisensory visual targets being more accurate than to unisensory auditory targets, but auditory saccades being more rapid (Corneil *et al.*, 2002). The pattern of auditory and visual unisensory accuracy is also consistent with a “best of both worlds” phenomenon: vision dominates near the midline where localisation acuity is higher than for auditory stimuli, and the reverse occurs in the periphery.

Where and how multisensory signals are integrated for decision-making is likely to be task and stimulus dependent (Bizley *et al.*, 2016). The improvement in performance observed for multisensory stimuli could arise through multiple mechanisms. It might be that by cueing cross-modal spatial attention to a particular region of space with the reference stimulus, performance is enhanced (Spence and Driver, 1997). It may be that early cross-modal integration of auditory and visual signals within auditory cortex (Bizley and King, 2008) enables the visual stimulus to improve the representation of the sound in auditory cortex. A final alternative is that separate auditory and visual estimates of the relative location of the reference and target sound might allow weighted integration at a later decision-making stage (Alais and Burr, 2004). While relating localisation acuity and accuracy is non-trivial (Moore *et al.*, 2008), an improved reference representation should facilitate improved performance: if there is reduced uncertainty in the reference location (i.e., lower variance) the decision as to whether the target is to the left or right of this distribution should be more accurate. The results of experiment 2, in which an AV reference stimulus did not enhance the ability of observers to discriminate the direction of a subsequent auditory target, is therefore most consistent with the final option: that the improvement in performance seen for multisensory stimuli results from the integration of separate auditory and visual decisions. Optimal integration models generate testable predictions about how auditory and visual information are combined such that their integration is determined by the variance of the underlying unisensory estimates (Ernst and Banks, 2002).
